# Hyeonggaeyeongyo-Tang for Treatment of Allergic and Nonallergic Rhinitis: A Prospective, Nonrandomized, Pre-Post Study

**DOI:** 10.1155/2016/9202675

**Published:** 2016-09-06

**Authors:** Min-Hee Kim, Jaewoong Son, Hae Jeong Nam, Seong-Gyu Ko, Inhwa Choi

**Affiliations:** ^1^Department of Clinical Korean Medicine, Graduate School, Kyung Hee University, Seoul, Republic of Korea; ^2^Department of Otorhinolaryngology of Korean Medicine, Kyung Hee University Hospital at Gangdong, Seoul, Republic of Korea; ^3^Department of Ophthalmology & Otorhinolaryngology, College of Korean Medicine, Kyung Hee University, Seoul, Republic of Korea; ^4^Department of Preventive Medicine, College of Korean Medicine, Kyung Hee University, Seoul, Republic of Korea

## Abstract

Hyeonggaeyeongyo-tang (HYT) is an ancient formula of oriental medicine traditionally used to treat rhinitis; however, clinical evidence has not yet been established. The aim of this study was to investigate the short-term and long-term efficacy and safety of HYT for chronic rhinitis. Adult subjects with chronic rhinitis symptoms were recruited. The subjects received HYT for 4 weeks and had follow-up period of 8 weeks. Any medicines used to treat nasal symptoms were not permitted during the study. The skin prick test was performed to distinguish the subjects with allergic rhinitis from those with nonallergic rhinitis. After treatment, the total nasal symptoms score and the Rhinoconjunctivitis Quality of Life Questionnaire score significantly improved in the whole subject group, in the allergic rhinitis group, and in the nonallergic rhinitis group, with no adverse events. This improvement lasted during a follow-up period of 8 weeks. Total IgE and eosinophil levels showed no significant difference after treatment in the allergic rhinitis group. HYT improved nasal symptoms and quality of life in patients with allergic rhinitis and nonallergic rhinitis. This is the first clinical study to evaluate the use of HYT to treat patients with rhinitis. This trial has been registered with the ClinicalTrials.gov Identifier NCT02477293.

## 1. Introduction

Rhinitis is defined by its clinical symptoms: rhinorrhea, nasal congestion, nasal itching, and sneezing. Anywhere from 10 to 40% of the population in industrialized countries has rhinitis based on epidemiologic surveys [1–4]. Chronic rhinitis (CR) is a chronic form of rhinitis and has been historically divided into allergic rhinitis (AR) and nonallergic rhinitis (NAR).

In Korea, several herbal medicines have been used to treat rhinitis. Hyeonggaeyeongyo-tang (HYT), also known as Keigai-rengyo-to or Jing Jie Lian Qiao Tang, is mixed herbal formula that has been used for hundreds of years in the treatment of rhinitis, rhinosinusitis, and acne. In an exploratory study, HYT was ranked first on the list of most commonly used herbal medications for treatment of allergic rhinitis in three Korean medical hospitals [5]. In another study, specialists in the Department of Otorhinolaryngology of Korean Medicine selected HYT as the third most preferred medicine to treat allergic rhinitis [6].

In animal studies, HYT has been shown to decrease the vascular permeability response to intradermal histamine and serotonin and to suppress a delayed type hypersensitivity response [7, 8]. Park and Hong reported that HYT has anti-inflammation effects for AR via the suppression of NF-*κ*B activation and iNOS production in BALB/c mice [9]. Hong et al. reported that HYT reduces infiltration of inflammatory cells and mast cells into the nasal cavity and reduces the levels of cytokines and leukocytes in the blood in an ovalbumin-induced AR model [10]. In a clinical study, HYT has shown effectiveness in the treatment of adult patients with acne vulgaris [11]. To our knowledge, no other clinical studies have evaluated HYT for nasal symptoms thus far.

We conducted a prospective, nonrandomized, single-armed, pre-post study of Korean adults with rhinitis. The aim of this study was to investigate the short-term and long-term efficacy and the safety of HYT treatment for allergic and nonallergic rhinitis.

## 2. Methods

### 2.1. Study Design

This prospective, nonrandomized, single-armed, pre-post study was conducted at the Department of Otorhinolaryngology of Korean Medicine at Kyung Hee University Hospital at Gangdong. The study's flow chart is shown in Table 1. This study was approved by the Institutional Review Board of Kyung Hee University Hospital at Gangdong (KHNMC-OH-IRB 2015-04-009). Written informed consent was obtained from all subjects prior to enrollment.

### 2.2. Subjects

A total of 40 subjects with CR were enrolled. The inclusion criteria were as follows: (1) age of 18–65 years, (2) presence of one or more nasal symptoms (rhinorrhea, nasal congestion, nasal itching, and sneezing) for more than 12 weeks, and (3) moderate-to-severe rhinitis (at least one of the following moderate abnormality conditions: sleep disturbance; limitations in daily activity, physical exercise, or leisure activity; work/school limitations; and discomfort from several symptoms) [12]. The exclusion criteria were as follows: (1) treatment with nasal/oral corticosteroids within the past month; nasal cromolyn or tricyclic antidepressants within the past two weeks; or nasal/oral decongestants, nasal/oral antihistamines, or antileukotrienes within the past week, (2) presence of hypertension (systolic ≥ 180 mmHg or diastolic ≥ 100 mmHg) or severe anemia (hemoglobin ≤ 10 g/dL (male) and 9 g/dL (female)), (3) presence of abnormal liver function (aspartate transaminase (AST) or alanine transaminase (ALT) ≥ 100 IU/L) or abnormal renal function (blood urea nitrogen (BUN) ≥ 30 mg/dL or creatinine ≥ 1.8 mg/dL (male) and 1.5 mg/dL (female)), (4) presence of neoplasm, severe systemic inflammation, or other systemic diseases that affect rhinitis, (5) history of drug allergy, (6) history of anaphylaxis for allergic tests, and (7) pregnancy or lactation.

### 2.3. Study Drug

Patients were treated with HYT extracted with water (Hanpoong Hyeonggaeyeongyo-tang granules, Hanpoong Pharm & Food Co., Ltd., Jeonju, Republic of Korea). It is a brown, bitter, herbal extract and was produced according to the Korean Good Manufacturing Practice (GMP) guidelines as permitted by the Korean Food & Drug Administration. HYT granules were sealed in opaque aluminum bags and administered to participants at doses of 3 g in accordance with standard guidelines for herbal prescription administration. The pharmacists instructed the subjects to dissolve HYT (3 g) from each package in water and take the solutions 30 minutes after each meal three times per day for 4 weeks. The composition of HYT is shown in Table 2. During the study, any medications that may have affected nasal symptoms (antihistamines, corticosteroids, anticholinergics, antileukotriene drugs, decongestants, tricyclic antidepressants, phenothiazines, nonsteroidal anti-inflammatory drugs, or Korean herbal medicines that were judged by the researchers to affect nasal symptoms) were not permitted. Subjects that reported using any of these medications were excluded from the study.

### 2.4. Allergic Skin Prick Test

The skin prick test was performed according to routine procedure. Eleven common aeroallergens (*Dermatophagoides farinae*,* Dermatophagoides pteronyssinus*, dog fur, cat fur, grass mixture, tree mixture, mugwort, ragweed,* Alternaria tenuis*,* Aspergillus fumigatus*, and cockroach), negative controls (50% glycerin saline), and positive controls (0.1% histamine phosphate) were used (Allergopharma GmbH & Co. KG, Reinbek, Germany). The subjects who showed a positive reaction to the skin prick test were identified as AR, while subjects who showed no reaction to the skin prick test were identified as NAR.

### 2.5. Efficacy Assessment

Any change in the TNSS was considered the primary efficacy variable, while any change in the RQLQ was considered a secondary efficacy variable. The TNSS evaluates symptoms of rhinorrhea, nasal congestion, nasal itching, and sneezing on a 4-point scale. The total score range is from 0 to 12, where 0 = no symptoms, 1 = mild symptom(s) (present but bearable), 2 = moderate symptom(s) (present and uncomfortable), and 3 = severe symptom(s) (unbearable). Before and after medication we examined total serum IgE and eosinophil count for subjects with AR.

### 2.6. Safety Assessment

Before and after medication we assessed levels of AST/ALT, BUN/creatinine, complete blood counts (CBC) including white blood cell (WBC), red blood cell (RBC), hemoglobin, hematocrit, and platelet, and erythrocyte sedimentation rate (ESR) to ensure the subjects' safety. Throughout the trial all adverse events were noted in subjects' reports or in the case report forms.

### 2.7. Statistical Analysis

All statistical analyses were performed using the SPSS 21 (IBM Inc., Armonk, NY, USA), and values are presented as means ± standard deviations. A repeated-measures ANOVA test with Bonferroni post hoc test was performed to evaluate the changes of TNSS and RQLQ scores throughout the 12 weeks. Independent* t*-tests were performed in order to analyze the intergroup analysis at each period. Paired* t*-tests were used to compare values before and after medication in each group. In all tests, a value of *p* < 0.05 was considered statistically significant.

## 3. Results

### 3.1. Subjects

A total of 47 patients were screened, and 40 subjects with CR were included in this study. Seven patients were excluded because they had mild nasal symptoms. During the active treatment period, 2 subjects withdrew from the study because of personal reasons. During the follow-up period, 3 subjects were excluded because they used other medicines. There were 16 subjects who were identified as AR after skin prick test. All AR subjects showed positive reaction to at least one or more persistent allergens. There were no significant differences in sex, age, baseline TNSS, each nasal symptom score, and baseline RQLQ score between the AR group and the NAR group (Table 3).

### 3.2. TNSS

**Figure 1:**
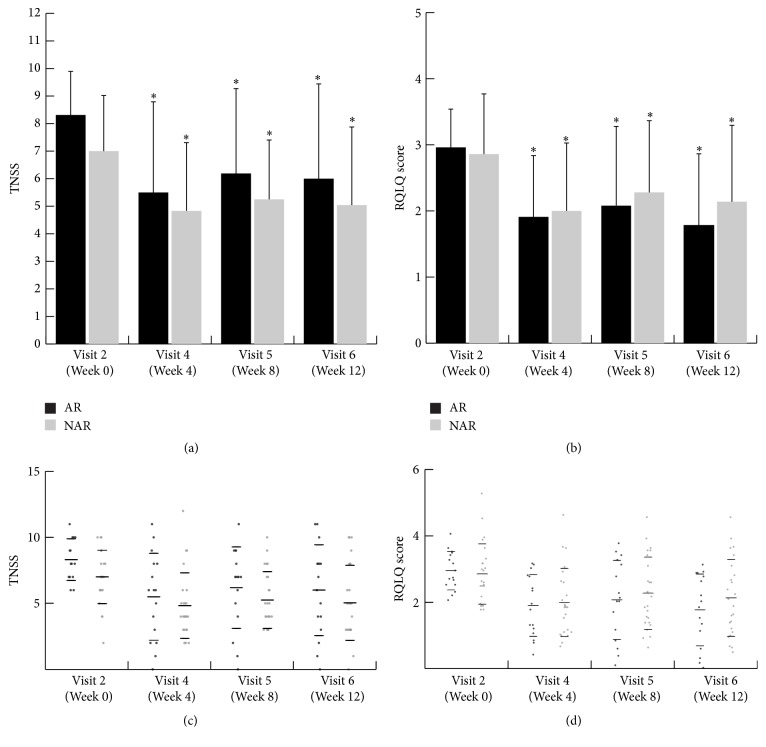
Effects of HYT on the AR group and the NAR group. (a and c) Mean TNSS at each visit. (b and d) Mean RQLQ score at each visit. ^*∗*^Significant difference (*p* < 0.05) compared with week 0 (baseline) in each group (repeated-measures ANOVA test and Bonferroni post hoc test). HYT, Hyeonggaeyeongyo-tang; AR, allergic rhinitis; NAR, nonallergic rhinitis; TNSS, total nasal symptom score; RQLQ, Rhinoconjunctivitis Quality of Life Questionnaire.

A statistically significant decrease in TNSS was observed after medication (week 4), and this decrease lasted for the follow-up period in the whole subject group (CR), the AR group, and the NAR group. There was no significant between-group difference at any time point (Table 4 and Figures 1(a) and 1(c)). All nasal symptoms, rhinorrhea, nasal congestion, nasal itching, and sneezing, showed significant improvement during treatment. There was no significant difference in any of the nasal symptoms between the AR group and the NAR group (Table 5).

### 3.3. RQLQ

The RQLQ showed a statistically significant decrease after medication and this improvement lasted for follow-up period in the whole subject group (CR), the AR group, and the NAR group. There was no significant between-group difference at any time point (Table 6 and Figures 1(b) and 1(d)).

### 3.4. Serum Total IgE and Eosinophil Count

There was no significant change in total serum IgE or eosinophil count after treatment in the AR group (Table 7).

### 3.5. Safety

There were no serious adverse events observed or reported during the study. The only minor adverse events observed during treatment were dyspepsia (*n* = 3) and xerostomia (*n* = 2), but these events disappeared during the follow-up period. There were statistically significant differences in BUN, RBC, hemoglobin, and hematocrit after treatment. However, all these changes were in normal ranges and there was no subject who showed abnormal value after treatment assessment (Table 8).

## 4. Discussion

CR is chronic form of rhinitis and has been classified as AR and NAR. Approximately 50% of CR patients are classified as having AR, and the others are classified as having NAR [13]. AR is characterized by a specific IgE response against relevant aeroallergens. NAR encompasses all forms of rhinitis in which a specific IgE response against relevant aeroallergens is absent.

The main medications currently used for allergic and nonallergic rhinitis are antihistamines, nasal steroids, nasal decongestants, and leukotriene receptor antagonists [14, 15]. In many cases of CR, symptoms are prolonged for years throughout all seasons; therefore, it is necessary to find the medicine which has no adverse effects for long-term medication and has long lasting effects. However, long-term use of many of the medications used for the treatment of CR can cause adverse effects. Antihistamines are the most widely used treatment for both AR and NAR. Antihistamines have limited efficacy in treating nasal congestion and commonly cause adverse effects such as sedation and weight gain [16, 17]. Nasal decongestants are useful for nasal obstruction, but using them for more than a week is not recommended because of adverse effects and drug tolerance [18]. For these reasons, traditional Chinese medicines which are made with natural herbs have recently gained much interest [19].

In this study, HYT improved nasal symptoms and quality of life in patients with AR and NAR after 4 weeks of medication, and these effects lasted 8 weeks after the end of medication. Each rhinitis patient has different symptoms based on his or her type of rhinitis and environmental factors. Some patients mainly have watery rhinorrhea and sneezing symptoms, while others mainly have nasal obstruction symptoms. In our study, both rhinorrhea and nasal obstruction were improved in patients with AR and NAR.

Other studies reported that HYT has anti-inflammation effects for AR by the suppression of NF-*κ*B activation and iNOS production in BALB/c mice [9]. In several previous studies, main components of HYT,* Schizonepeta tenuifolia*,* Forsythia* fruit,* Saposhnikovia divaricata*, and* Bupleurum falcatum*, were observed to have antioxidant and anti-inflammatory activities [20–23]. These antioxidant and anti-inflammatory activities could be the reason of the effect of HYT in patients with AR and NAR.

In a previous animal study, HYT had antiallergic effects, inhibiting the increase of the levels of IL-4, IL-13, leukemia inhibitory factor (LIF), eosinophils, neutrophils, monocytes, basophils, lymphocytes, and WBC in ovalbumin-induced AR model [10]. Based on previous studies, we hypothesized that IgE and eosinophils will be suppressed in patients with AR; however, current study showed no decrease in IgE and eosinophils in AR patients. By referring to previous clinical study that reported changes of serum IgE, cytokines, IL-4 stimulated prostaglandin E2 (PGE2), and polymorphonuclear leukocyte (PMN) after 12-week administration of oriental herbal medicine, duration of medication needed to be longer in our study to observe the changes of serum IgE and eosinophils [24, 25].

There were statistically significant differences in BUN, RBC, hemoglobin, and hematocrit after treatment. However, all these changes were in normal ranges and there was no subject who showed abnormal value after treatment assessment. A clinical study with long-term medication for at least 8 weeks and the safety assessment would be necessary to confirm the long-term safety of HYT and to observe antiallergic effects in laboratory tests.

Our study has several limitations. First, this study is pre-post study that was conducted with a nonrandomized and no-control-group design. Second, our sample size was relatively small. Third, the medication period was relatively short for safety assessment.

Despite these limitations, this study has a meaning as the first clinical study for HYT in CR. From this study, we suggest that HYT could be investigated as a medicine for patients with AR and NAR. A clinical study with a randomized, double-blind, placebo-controlled design, larger sample size, and long-term medication should be performed to obtain more accurate knowledge about HYT treatment for rhinitis and to observe its mechanism.

## Figures and Tables

**Table 1:** Study's flow chart.

Stage	Screening	Active treatment (4-week)			Follow-up (8-week)	
Visit	1	2	3	4	5	6
Weeks	−1	0	2	4	8	12

Informed consent	○					
Inclusion/exclusion criteria						
Skin prick test		○				
TNSS & RQLQ		○	○	○	○	○
Total IgE & eosinophil count^†^		○		○		
Vital sign	○	○	○	○	○	○
Laboratory tests for safety assessment^*∗*^	○			○		
Adverse event			○	○	○	○

^†^Only for AR subjects; ^*∗*^complete blood cell counts, levels of aspartate transaminase, alanine transaminase, blood urea nitrogen, creatinine, and erythrocyte sedimentation rate.

TNSS, total nasal symptom score; RQLQ, Rhinoconjunctivitis Quality of Life Questionnaire; IgE, immunoglobulin E.

**Table 2:** The composition of herbal medicines in Hyeonggaeyeongyo-tang.

Component	Volume (g)
*Schizonepeta tenuifolia*	0.50
*Forsythia* fruit	0.50
Saposhnikovia radix	0.50
Angelicae gigantis radix	0.50
Cnidii rhizome	0.50
Paeoniae radix alba	0.50
Glycyrrhizae radix	0.50
Bupleuri radix	0.50
Ponciri fruit	0.50
Scutellariae radix	0.50
Gardeniae fruit	0.50
Angelicae dahuricae radix	0.83
Platycodi radix	0.83

**Table 3:** Demographic characteristics of the study population.

Number of subjects	40
Male/female (*n*)	15/25
Age (mean ± SD, years)	38.68 ± 12.86
Dropouts	
Personal reasons	2
Use of other medications	3
AR/NAR group (*n*)	16/24
Persistent allergens	
*Dermatophagoides farinae*	15
*Dermatophagoides pteronyssinus*	15
Cockroach	1
Dog fur	0
Cat fur	5
*Aspergillus fumigatus*	2
*Alternaria tenuis*	3
Seasonal allergens	
Grass mixture	1
Mugwort	6
Tree mixture	3
Ragweed	1

SD, standard deviations; AR, allergic rhinitis; NAR, nonallergic rhinitis.

**Table 4:** Mean TNSS from baseline to week 12.

	Week 0	Week 4	Week 8	Week 12
All subjects	7.53 ± 1.95	5.10 ± 2.81^*∗*^	5.63 ± 2.57^*∗*^	5.43 ± 3.09^*∗*^
AR	8.31 ± 1.58	5.50 ± 3.29^*∗*^	6.19 ± 3.08^*∗*^	6.00 ± 3.44^*∗*^
NAR	7.00 ± 2.02	4.83 ± 2.48^*∗*^	5.25 ± 2.15^*∗*^	5.04 ± 2.84^*∗*^

Mean ± standard deviation, repeated-measures ANOVA test, and Bonferroni post hoc test.

^*∗*^Significant difference (*p* < 0.05) compared with week 0 (baseline) in each group (no significant difference between week 4, week 8, and week 12 in each group).

TNSS, total nasal symptom score; AR, allergic rhinitis; NAR, nonallergic rhinitis.

**Table 5:** Nasal symptom score from baseline to week 4 in AR and NAR group.

	Week 0	Week 4	*p* value
Rhinorrhea			
AR	2.06 ± 0.93	1.44 ± 1.09	0.036^*∗*^
NAR	1.88 ± 0.90	1.29 ± 0.95	0.001^*∗*^
Nasal congestion			
AR	1.69 ± 0.95	1.00 ± 0.89	0.029^*∗*^
NAR	1.92 ± 0.88	1.42 ± 0.72	0.043^*∗*^
Nasal itching			
AR	2.50 ± 0.63	1.56 ± 1.03	0.001^*∗*^
NAR	1.54 ± 0.98	0.92 ± 1.02	0.002^*∗*^
Sneezing			
AR	2.06 ± 0.44	1.50 ± 0.97	0.045^*∗*^
NAR	1.67 ± 0.96	0.96 ± 0.86	0.001^*∗*^

Mean ± standard deviation and paired *t*-test.

^*∗*^Significant difference (*p* < 0.05) between week 0 (baseline) and week 4 in each group.

TNSS, total nasal symptom score; AR, allergic rhinitis; NAR, nonallergic rhinitis.

**Table 6:** Mean RQLQ scores from baseline to week 12.

	Week 0	Week 4	Week 8	Week 12
All subjects	2.90 ± 0.79	1.96 ± 0.98^*∗*^	2.20 ± 1.12^*∗*^	2.00 ± 1.13^*∗*^
AR	2.96 ± 0.58	1.91 ± 0.93^*∗*^	2.08 ± 1.19^*∗*^	1.78 ± 1.08^*∗*^
NAR	2.86 ± 0.91	2.00 ± 1.03^*∗*^	2.28 ± 1.09^*∗*^	2.14 ± 1.16^*∗*^

Mean ± standard deviation, repeated-measures ANOVA test, and Bonferroni post hoc test.

^*∗*^Significant difference (*p* < 0.05) compared with week 0 (baseline) in each group (no significant difference between week 4, week 8, and week 12 in each group).

RQLQ, Rhinoconjunctivitis Quality of Life Questionnaire; AR, allergic rhinitis; NAR, nonallergic rhinitis.

**Table 7:** Total serum IgE and eosinophil count in the AR group.

	Week 0	Week 4	*p* value
Total IgE (IU/mL)	287.93 ± 321.26	233.21 ± 183.23	0.486
Eosinophil count (/*μ*L)	271.25 ± 211.03	281.25 ± 244.29	0.847

Mean ± standard deviation and paired *t*-test.

IgE, immunoglobulin E; AR; allergic rhinitis.

**Table 8:** The laboratory tests for safety assessment.

	Normal ranges	Week 0	Week 4	*p* value
AST	0~40 (U/L)	21.50 ± 6.84	24.45 ± 14.47	0.148
ALT	0~40 (U/L)	18.84 ± 11.39	22.95 ± 22.43	0.167
BUN	8~23 (mg/dL)	13.24 ± 3.94	11.73 ± 3.26	0.005^*∗*^
Creatinine	0.6~1.2 (mg/dL)	0.74 ± 0.15	0.73 ± 0.16	0.354
CBC				
WBC	4.0~10.0 (×10^3^/*μ*L)	6.76 ± 1.33	6.45 ± 1.38	0.116
RBC	4.2~6.3 (×10^6^/*μ*L)	4.66 ± 0.51	4.57 ± 0.48	0.019^*∗*^
Hemoglobin	13~17 (g/dL)	13.93 ± 1.60	13.64 ± 1.53	0.002^*∗*^
Hematocrit	36~48 (%)	41.64 ± 4.24	40.87 ± 4.01	0.011^*∗*^
Platelet	150~350 (×10^3^/*μ*L)	261.95 ± 46.21	261.49 ± 51.24	0.890
ESR	~20 (mm/h)	12.62 ± 8.97	11.49 ± 8.30	0.290

Mean ± standard deviation and paired *t*-test.

^*∗*^Significant difference (*p* < 0.05) between week 0 (baseline) and week 4.

AST, aspartate transaminase; ALT, alanine transaminase; BUN, blood urea nitrogen; CBC, complete blood count; WBC, white blood cell; RBC, red blood cell; ESR, erythrocyte sedimentation rate.
